# Supplementary material for: Precision and Accuracy of Field Versus Laboratory Bioassay Insecticide Efficacy for the Control of Immature Bemisia tabaci
Source: Insects. 2023 Jul 18;14(7):645. doi: 10.3390/insects14070645 (PMC10380993; doi:10.3390/insects14070645)
Supplement: Supplementary file 1 [file insects-14-00645-s001.zip › insects-2444339-supplementary.pdf]

Supl. Table S1. Laboratory and field counts (± SD) of *Bemisia tabaci* immatures at seven days after treatment with different insecticides, Coastal Plain Experiment Station, Tifton, GA, 2021-2022.

Crop	Treatment	Eggs		Nymphs	
		Bioassay[1]	Field	Bioassay	Field
		Avg. ± SD dif[2]	Avg. ± SD	Avg. ± SD	Avg. ± SD
Squash #1 2021 n=88	Check	47.75 ± 18.07 a	14.18 ± 8.94 a	60.75 ± 22.71 a	4.73 ± 3.49 a
	Pyriproxyfen	45.75 ± 10.26 a	0.95 ± 0.66 b	1.25 ± 0.75 b	0.30 ± 0.60 a
	Clothianidin	7.25 ± 3.92 b	2.18 ± 3.50 b	15.25 ± 9.12 ab	0.67 ± 0.94 a
	Spiromesifen	17.25 ± 6.33 b	1.20 ± 1.28 b	10.50 ± 8.62 b	3.55 ± 6.33 a
	Flonicamid	6.50 ± 5.55 b	0.40 ± 0.80 b	13.25 ± 13.25 b	1.40 ± 1.41 a
	Sulfoxaflor	6.25 ± 3.17 b	1.40 ± 1.49 b	10.00 ± 3.63 b	0.95 ± 1.77 a
	Imidacloprid	5.75 ± 2.95 b	1.95 ± 3.77 b	4.25 ± 2.66 b	1.10 ± 1.24 a
	Acetamiprid	0.75 ± 0.75 b	1.20 ± 1.25 b	1.50 ± 0.87 b	0.48 ± 0.76 a
	Cyantraniliprole	1.50 ± 0.96 b	2.60 ± 3.27 b	1.00 ± 0.71 b	2.00 ± 1.10 a
	Flupyradifurone	0.75 ± 0.48 b	0.40 ± 0.54 b	1.50 ± 0.96 b	0.95 ± 1.00 a
	Dinotefuran	5.75 ± 5.11 b	0.85 ± 0.81 b	0.75 ± 0.75 b	2.70 ± 3.04 a
Cucumber #1 2021 n=88	Check	8.00 ± 1.47 a	13.00 ± 2.67 a	3.75 ± 1.75 a	6.25 ± 4.27 a
	Pyriproxyfen	0.25 ± 0.25 b	2.70 ± 0.90 b	0.00 ± 0.00 b	0.50 ± 1.00 b
	Clothianidin	2.00 ± 1.08 b	1.65 ± 0.50 b	0.50 ± 0.50 b	1.25 ± 1.50 b
	Spiromesifen	0.00 ± 0.00 b	2.10 ± 0.99 b	0.00 ± 0.00 b	1.25 ± 0.50 b
	Flonicamid	1.75 ± 1.75 b	2.15 ± 0.82 b	2.50 ± 2.50 a	1.00 ± 0.82 b
	Sulfoxaflor	1.25 ± 0.75 b	2.95 ± 1.34 b	0.25 ± 0.25 b	1.25 ± 0.50 b
	Imidacloprid	0.00 ± 0.00 b	0.75 ± 0.66 b	0.00 ± 0.00 b	1.25 ± 0.96 b
	Acetamiprid	0.00 ± 0.00 b	2.15 ± 0.60 b	0.00 ± 0.00 b	0.75 ± 0.96 b
	Cyantraniliprole	0.00 ± 0.00 b	0.95 ± 0.68 b	0.00 ± 0.00 b	1.00 ± 1.15 b
	Flupyradifurone	0.00 ± 0.00 b	1.15 ± 1.52 b	0.00 ± 0.00 b	0.75 ± 0.96 b
	Dinotefuran	0.00 ± 0.00 b	0.74 ± 1.11 b	0.00 ± 0.00 b	0.50 ± 0.58 b
Squash #2 2021 n=88	Check	63.25 ± 22.46 a	10.25 ± 3.97 a	96.75 ± 39.82 a	4.85 ± 2.50 a
	Pyriproxyfen	21.50 ± 6.51 abc	1.30 ± 1.25 c	6.00 ± 2.27 b	0.85 ± 1.14 b
	Clothianidin	25.00 ± 4.95 ab	5.95 ± 2.58 ab	31.50 ± 5.06 ab	2.30 ± 0.66 ab
	Spiromesifen	14.50 ± 7.69 bc	5.60 ± 2.92 ab	19.50 ± 14.66 b	2.00 ± 2.02 ab
	Flonicamid	16.00 ± 2.48 bc	2.00 ± 0.86 bc	20.75 ± 7.64 b	2.60 ± 2.66 ab
	Sulfoxaflor	9.00 ± 3.74 bc	4.15 ± 1.10 bc	13.00 ± 4.53 b	2.75 ± 0.97 ab
	Imidacloprid	8.00 ± 2.35 bc	2.25 ± 1.49 bc	1.50 ± 0.96 b	0.65 ± 0.79 b
	Acetamiprid	5.50 ± 2.25 bc	3.50 ± 1.54 bc	10.75 ± 5.47 b	0.80 ± 0.59 b

	Cyantraniliprole	2.00 ± 0.71 bc	1.25 ± 0.41 c	3.25 ± 1.44 b	1.95 ± 1.44 ab
	Flupyradifurone	6.25 ± 1.80 bc	4.60 ± 1.98 abc	14.25 ± 5.07 b	1.80 ± 1.34 ab
	Dinotefuran	1.50 ± 0.65 c	1.15 ± 0.91 c	2.00 ± 2.00 b	1.10 ± 0.82 ab
Cucumber #2 2021 n=88	Check	81.00 ± 11.35 a	3.25 ± 1.06 a	125.75 ± 30.32 a	0.75 ± 0.57 a
	Pyriproxyfen	50.50 ± 3.07 ab	1.80 ± 1.07 abc	7.00 ± 2.16 cd	0.60 ± 0.54 a
	Clothianidin	30.75 ± 5.31 bc	0.25 ± 0.10 c	73.50 ± 15.55 ab	0.15 ± 0.30 a
	Spiromesifen	12.25 ± 3.57 cd	1.30 ± 0.50 abc	28.75 ± 10.01 bcd	0.35 ± 0.25 a
	Flonicamid	31.75 ± 4.53 bc	0.50 ± 0.12 c	41.50 ± 6.30 bc	0.10 ± 0.12 a
	Sulfoxaflor	17.50 ± 2.99 cd	1.80 ± 0.67 abc	22.75 ± 4.09 bcd	0.40 ± 0.28 a
	Imidacloprid	22.25 ± 4.61 cd	0.40 ± 0.28 c	39.75 ± 8.56 bc	0.20 ± 0.23 a
	Acetamiprid	15.25 ± 3.09 cd	2.70 ± 1.89 ab	22.00 ± 3.63 bcd	0.65 ± 0.50 a
	Cyantraniliprole	7.25 ± 2.66 de	0.80 ± 0.52 bc	16.25 ± 7.49 cd	0.15 ± 0.19 a
	Flupyradifurone	13.50 ± 4.37 cd	0.40 ± 0.43 c	17.50 ± 6.99 cd	0.15 ± 0.19 a
	Dinotefuran	1.00 ± 1.00 e	0.55 ± 0.62 c	3.25 ± 3.25 d	0.15 ± 0.19 a
Squash #3 2022 n=88	Check	79.25 ± 33.04 abc	360.55 ± 145.01 a	58.00 ± 34.09 a	159.45 ± 37.48 a
	Pyriproxyfen	103.50 ± 15.76 a	276.80 ± 103.57 a	1.25 ± 0.63 b	103.40 ± 21.79 a
	Clothianidin	62.75 ± 32.19 abcd	243.15 ± 80.52 a	45.75 ± 20.05 a	120.40 ± 29.46 a
	Spiromesifen	17.50 ± 10.52 bcd	223.65 ± 73.74 a	17.50 ± 5.87 ab	126.00 ± 17.39 a
	Flonicamid	86.25 ± 26.88 ab	247.55 ± 90.18 a	30.75 ± 12.50 ab	132.00 ± 18.51 a
	Sulfoxaflor	4.00 ± 1.58 d	285.00 ± 134.11 a	0.25 ± 0.25 b	125.35 ± 10.58 a
	Imidacloprid	23.75 ± 7.86 abcd	240.80 ± 86.67 a	39.50 ± 8.07 a	134.95 ± 21.76 a
	Acetamiprid	5.25 ± 0.48 cd	229.30 ± 26.67 a	0.75 ± 0.48 b	124.35 ± 32.18 a
	Cyantraniliprole	1.75 ± 1.03 d	230.70 ± 35.72 a	0.00 ± 0.00 b	111.25 ± 20.83 a
	Flupyradifurone	6.50 ± 3.18 cd	164.20 ± 76.02 a	1.50 ± 0.65 b	103.10 ± 33.38 a
	Dinotefuran	1.25 ± 0.48 d	169.10 ± 86.46 a	0.25 ± 0.25 b	100.90 ± 21.66 a
Cucumber #3 2022 n=88	Check	31.25 ± 3.47 a	115.80 ± 53.99 a	69.75 ± 6.46 a	13.45 ± 3.04 a
	Pyriproxyfen	6.75 ± 1.70 bc	89.10 ± 22.08 a	1.25 ± 0.48 f	6.75 ± 6.88 a
	Clothianidin	11.00 ± 1.87 b	100.65 ± 39.93 a	29.25 ± 4.82 b	8.70 ± 4.45 a
	Spiromesifen	7.25 ± 0.95 bc	60.95 ± 32.27 a	15.75 ± 1.25 bcd	5.45 ± 4.74 a
	Flonicamid	8.75 ± 1.44 bc	89.55 ± 30.92 a	25.00 ± 3.85 bc	11.95 ± 5.94 a
	Sulfoxaflor	6.75 ± 1.31 bc	107.35 ± 75.55 a	13.75 ± 2.39 cd	5.80 ± 3.70 a
	Imidacloprid	11.00 ± 1.58 b	99.20 ± 58.76 a	28.25 ± 4.96 bc	8.05 ± 5.32 a
	Acetamiprid	7.00 ± 1.47 bc	58.65 ± 17.24 a	9.75 ± 1.60 de	3.65 ± 1.91 a
	Cyantraniliprole	7.75 ± 1.89 bc	28.10 ± 12.69 a	8.75 ± 1.03 de	4.40 ± 4.08 a
	Flupyradifurone	7.75 ± 1.38 bc	60.95 ± 20.47 a	14.50 ± 3.71 bcd	6.05 ± 4.42 a
	Dinotefuran	3.25 ± 0.48 c	74.45 ± 43.11 a	3.00 ± 0.71 ef	3.80 ± 2.41 a
Squash #4	Check	68.25 ± 18.43 a	508.00 ± 167.48 a	21.75 ± 5.59 a	189.80 ± 225.66 a

2022 n=88	Pyriproxyfen	51.50 ± 11.99 abc	727.33 ± 340.85 a	9.50 ± 1.85 abcd	527.73 ± 493.63 a
	Clothianidin	44.75 ± 10.82 abc	359.93 ± 158.37 a	15.75 ± 1.65 ab	121.20 ± 125.28 a
	Spiromesifen	46.00 ± 12.46 abc	495.40 ± 202.52 a	17.00 ± 3.03 ab	282.20 ± 212.24 a
	Flonicamid	55.75 ± 12.01 ab	418.05 ± 171.49 a	20.00 ± 4.71 a	196.65 ± 269.43 a
	Sulfoxaflor	47.25 ± 10.81 abc	442.20 ± 84.29 a	15.00 ± 2.35 ab	157.35 ± 136.27 a
	Imidacloprid	38.00 ± 11.48 abcd	589.70 ± 238.75 a	13.25 ± 3.35 abc	239.40 ± 121.19 a
	Acetamiprid	17.25 ± 5.45 abc	433.10 ± 301.28 a	5.50 ± 0.96 bcd	205.90 ± 336.38 a
	Cyantraniliprole	7.50 ± 1.44 d	556.25 ± 238.85 a	3.50 ± 0.65 cd	375.35 ± 274.01 a
	Flupyradifurone	11.25 ± 2.02 d	383.05 ± 143.75 a	4.00 ± 1.08 cd	201.80 ± 158.47 a
	Dinotefuran	6.75 ± 1.89 d	429.13 ± 90.70 a	3.00 ± 1.47 d	303.47 ± 21.35 a
Cucumber #4 2022 n=88	Check	67.75 ± 12.99 a	183.80 ± 57.99 a	14.50 ± 1.55 a	54.25 ± 39.21 a
	Pyriproxyfen	55.75 ± 14.63 ab	73.25 ± 18.38 b	5.50 ± 2.22 bc	13.30 ± 5.33 b
	Clothianidin	50.00 ± 13.07 abc	123.35 ± 84.92 ab	10.25 ± 1.38 ab	31.80 ± 15.62 ab
	Spiromesifen	39.25 ± 1.60 abc	94.00 ± 30.93 ab	7.50 ± 0.96 abc	22.26 ± 13.93 ab
	Flonicamid	55.50 ± 6.65 a	80.05 ± 40.05 ab	11.00 ± 1.83 ab	19.65 ± 8.96 ab
	Sulfoxaflor	45.25 ± 9.58 abc	123.10 ± 48.50 ab	8.00 ± 1.83 abc	23.30 ± 10.77 ab
	Imidacloprid	33.00 ± 6.35 abc	87.70 ± 27.99 ab	6.75 ± 1.03 abc	18.25 ± 3.79 ab
	Acetamiprid	29.75 ± 12.80 abc	104.40 ± 39.62 ab	8.25 ± 0.63 abc	17.80 ± 2.93 ab
	Cyantraniliprole	13.50 ± 2.50 c	80.55 ± 20.29 ab	2.25 ± 0.85 c	14.05 ± 6.66 b
	Flupyradifurone	23.25 ± 4.82 abc	99.05 ± 23.74 ab	3.00 ± 0.71 c	9.25 ± 3.99 b
	Dinotefuran	14.00 ± 2.65 bc	62.15 ± 6.37 b	2.50 ± 1.04 c	12.60 ± 6.22 b

[1] Bioassay counts after one week following adult oviposition for 48 h in a treated cotton leaf

[2] Means followed by the same letter within each column are not significantly different (*P*<0.05, Tukey`s test). To account for natural null values in the dataset, a square root transformation (√x+1) was performed prior to conducting the analysis.

Supl. Table S2. Precision (CV%) and accuracy (RMSE and MAE) of insecticide efficacy estimates on *Bemisia tabaci* immatures at seven days after treatment with different insecticides, Coastal Plain Experiment Station, Tifton, GA, 2021-2022.

Crop	Treatment	Eggs				Nymphs			
		Lab CV (%)	Field CV (%)	RMSE	MAE	Lab	Field	RMSE	MAE
Squash #1 n=88	Check	37.85	63.10	0.130	0.100	37.38	73.83	0.223	0.188
	Pyriproxyfen	22.42	69.56	0.477	0.446	60.00	200.00	0.035	0.021
	Clothianidin	54.12	160.42	0.159	0.127	59.82	141.42	0.098	0.071
	Spiromesifen	36.69	106.28	0.161	0.137	82.07	178.18	0.357	0.189
	Flonicamid	85.31	200.00	0.126	0.083	100.00	101.02	0.115	0.091
	Sulfoxaflor	50.75	106.27	0.068	0.056	36.29	186.23	0.115	0.093
	Imidacloprid	51.38	193.22	0.136	0.103	62.53	112.57	0.069	0.051
	Acetamiprid	100.00	104.33	0.053	0.037	57.74	157.42	0.047	0.029
	Cyantraniliprole	63.83	125.93	0.124	0.081	70.71	54.77	0.159	0.146
	Flupyradifurone	63.83	135.40	0.120	0.079	63.83	105.09	0.093	0.063
	Dinotefuran	88.79	94.85	0.094	0.062	100.00	112.58	0.279	0.202
Cucumbe r #1 n=88	Check	18.40	20.56	0.199	0.177	46.67	68.35	0.615	0.546
	Pyriproxyfen	100.00	33.40	0.169	0.162	.	200.00	.	.
	Clothianidin	54.01	30.30	0.159	0.112	100.00	120.00	0.087	0.054
	Spiromesifen	.	46.98	.	.	.	40.00	.	.
	Flonicamid	100.00	38.26	0.236	0.197	100.00	81.65	0.468	0.292
	Sulfoxaflor	60.00	45.44	0.108	0.088	100.00	40.00	0.079	0.079
	Imidacloprid	.	88.11	.	.	.	76.59	.	.
	Acetamiprid	.	27.78	.	.	.	127.66	.	.
	Cyantraniliprole	.	71.65	.	.	.	115.47	.	.
	Flupyradifurone	.	131.97	.	.	.	127.66	.	.
	Dinotefuran	.	148.27	.	.	.	115.47	.	.
Squash #2 n=88	Check	35.51	38.78	0.432	0.394	41.16	51.55	0.218	0.162
	Pyriproxyfen	30.29	96.08	0.134	0.105	37.88	133.62	0.140	0.097
	Clothianidin	19.80	43.43	0.193	0.160	16.06	28.84	0.152	0.147
	Spiromesifen	53.01	52.16	0.235	0.227	75.18	101.00	0.215	0.167
	Flonicamid	15.52	43.21	0.040	0.031	36.83	94.94	0.358	0.283
	Sulfoxaflor	41.57	26.51	0.200	0.183	34.83	35.32	0.307	0.298
	Imidacloprid	29.32	66.16	0.096	0.073	63.83	121.46	0.113	0.078

	Acetamiprid	40.99	44.14	0.189	0.172	50.86	73.60	0.062	0.052
	Cyantraniliprole	35.36	32.99	0.063	0.061	44.19	73.66	0.284	0.241
	Flupyradifurone	28.75	43.04	0.144	0.115	35.60	74.26	0.060	0.049
	Dinotefuran	43.03	79.54	0.080	0.072	100.00	74.97	0.156	0.135
Cucumber #2 n=88	Check	14.01	32.71	0.196	0.172	24.11	76.59	0.151	0.127
	Pyriproxyfen	6.08	59.49	0.184	0.140	30.86	90.27	0.439	0.342
	Clothianidin	17.28	40.00	0.248	0.238	21.15	200.00	0.274	0.255
	Spiromesifen	29.13	38.72	0.176	0.155	34.82	71.90	0.100	0.082
	Flonicamid	14.28	23.09	0.203	0.195	15.19	115.47	0.141	0.134
	Sulfoxaflor	17.06	37.41	0.242	0.210	17.98	70.71	0.189	0.173
	Imidacloprid	20.71	70.71	0.129	0.127	21.53	115.47	0.100	0.082
	Acetamiprid	20.28	70.14	0.515	0.419	16.49	76.92	0.386	0.330
	Cyantraniliprole	36.66	64.55	0.148	0.109	46.08	127.66	0.051	0.036
	Flupyradifurone	32.36	108.01	0.321	0.280	39.97	127.66	0.546	0.524
	Dinotefuran	100.00	112.57	0.154	0.105	100.00	127.66	0.113	0.078
Squash #3 n=88	Check	41.69	40.22	0.246	0.160	58.77	23.51	0.616	0.529
	Pyriproxyfen	15.22	37.42	0.254	0.189	50.33	21.08	0.536	0.527
	Clothianidin	51.30	33.11	0.420	0.395	43.83	24.47	0.381	0.335
	Spiromesifen	60.14	32.97	0.356	0.321	33.52	13.80	0.556	0.541
	Flonicamid	31.16	36.43	0.355	0.266	40.65	14.02	0.534	0.489
	Sulfoxaflor	39.53	47.05	0.583	0.537	100.00	8.44	0.648	0.647
	Imidacloprid	33.08	35.99	0.356	0.311	20.42	16.13	0.452	0.449
	Acetamiprid	9.12	11.63	0.420	0.418	63.83	25.88	0.654	0.638
	Cyantraniliprole	58.90	15.48	0.450	0.445	.	18.73	.	.
	Flupyradifurone	48.85	46.30	0.124	0.097	43.03	32.38	0.206	0.152
	Dinotefuran	38.30	51.13	0.361	0.327	100.00	21.47	0.529	0.520
Cucumber #3 n=88	Check	19.17	31.55	0.437	0.402	10.72	72.28	0.287	0.215
	Pyriproxyfen	26.24	25.09	0.253	0.237	40.32	40.06	0.458	0.341
	Clothianidin	26.14	68.84	0.288	0.185	13.43	49.11	0.292	0.262
	Spiromesifen	4.08	32.90	0.162	0.151	12.77	62.58	0.256	0.186
	Flonicamid	11.99	50.03	0.205	0.188	16.60	43.15	0.481	0.377
	Sulfoxaflor	21.17	39.40	0.465	0.321	22.82	46.24	0.260	0.219
	Imidacloprid	19.25	31.92	0.319	0.237	15.27	20.78	0.323	0.228
	Acetamiprid	43.01	37.95	0.133	0.096	7.63	16.46	0.105	0.079
	Cyantraniliprole	18.52	25.19	0.139	0.131	37.95	47.39	0.235	0.172
	Flupyradifurone	20.73	23.97	0.316	0.289	23.57	45.62	0.155	0.092

	Dinotefuran	18.90	10.25	0.314	0.271	41.63	49.33	0.198	0.165
Squash #4 n=88	Check	27.00	32.97	0.359	0.341	25.69	118.90	0.636	0.517
	Pyriproxyfen	23.28	46.86	0.352	0.331	19.46	93.54	0.474	0.388
	Clothianidin	24.18	44.00	0.210	0.210	10.49	103.37	0.397	0.386
	Spiromesifen	27.09	40.88	0.121	0.109	17.81	75.21	0.332	0.313
	Flonicamid	21.54	41.02	0.136	0.112	23.54	137.01	0.542	0.409
	Sulfoxaflor	22.88	19.06	0.213	0.204	15.63	86.60	0.368	0.305
	Imidacloprid	30.22	40.49	0.460	0.364	25.29	50.62	0.321	0.191
	Acetamiprid	31.61	69.57	0.430	0.328	17.41	163.37	0.324	0.254
	Cyantraniliprole	19.25	42.94	0.542	0.493	18.44	73.00	0.350	0.269
	Flupyradifurone	17.92	37.53	0.211	0.168	27.00	78.53	0.124	0.104
	Dinotefuran	27.96	21.14	0.325	0.278	49.07	7.04	0.181	0.152
Cucumber #4 n=88	Check	19.17	31.55	0.434	0.413	10.72	72.28	0.503	0.467
	Pyriproxyfen	26.24	25.09	0.437	0.417	40.32	40.06	0.247	0.206
	Clothianidin	26.14	68.84	0.455	0.395	13.43	49.11	0.216	0.197
	Spiromesifen	4.08	32.90	0.115	0.106	12.77	62.58	0.232	0.164
	Flonicamid	11.99	50.03	0.305	0.279	16.60	43.15	0.499	0.479
	Sulfoxaflor	21.17	39.40	0.069	0.053	22.82	46.24	0.252	0.244
	Imidacloprid	19.25	31.92	0.138	0.125	15.27	20.78	0.171	0.159
	Acetamiprid	43.01	37.95	0.322	0.287	7.63	16.46	0.246	0.242
	Cyantraniliprole	18.52	25.19	0.212	0.184	37.95	47.39	0.099	0.091
	Flupyradifurone	20.73	23.97	0.000	0.000	23.57	45.62	0.000	0.000
	Dinotefuran	18.90	10.25	0.116	0.103	41.63	49.33	0.151	0.135
